# Ancient Methods of Treatment

**Published:** 1906-12-29

**Authors:** 


### Ancient Methods of Treatment.

### CUTTING FOR STONE.

Everyone who has read Pepys' Diary knows of the feast by which he celebrated annually the day on which he was successfully cut for stone, and Mr. D'Arcy Power, the Honorary Treasurer of the Samuel Pepys Club, has shown * that Pepys was operated upon by Mr. Thomas Hollier, who was "surgeon for the stone" at St. Bartholomew's Hospital. The exact method adopted was in all probability identical with that described by James Cooke, "lover of physic and chyrurgery," who published his "Mellificium Chirurgiæ" in 1648. The directions for lithotomy are thus given: "The spring time is best for the operation and it is more safely performed on young than old"—Pepys was twenty-six when he was cut for the stone—"Have in readiness Catheters, Probes, Conductor, Itinerarium, specula, pincers, small hooks of all sizes, Astringent powders, Rowlers, Spunges and Cordialls. Let the patient be placed on a firm table with a sheet many times doubled laid under his buttocks and a pillow under his loynes and back, so that hee may lie half upright: with his thighs lifted up and his legs and heels drawn back to his hips: then having a long strong roller (bandage) of four fingers broad use it thus, let it rolled at both ends, let an Attendant hold one side of the roller very hard in the hinder part of the patient's neck and then goe with the other end over the patient's left armhole and under the left arm towards the right hip and over the fore part of the thigh, whence carry it below the knee, and thence bring it again to the externall part of the thigh and so to the sole of the foot and thence again above the thigh and so under the knee and thence again upwards towards the loynes; then go with it towards the left armhole, that so thou mayest bring it from under the left arm to the neck, where thou shalt deliver that end of the roller to be held by the attendant and taking the end which hee holds, thou shalt bring it over the right armpit first forward and then backward under the arms towards the left thigh, that both ends of the roller may meet crosswayes upon the back, whence thou shalt carry it above the hip and thigh, downwards to the knee, and above the shins, thigh and under the sole of the foot and so again to the hip and over the loynes to the right armhole, after the form used on the left side that both the ends of the roller may be knit together upon the neck surely.

"Being thus bound have two strong men on each side of him, two whereof may hold him by the knees and feet and two by the armholes and hands. After this let the chirurgion thrust in his Directory (staff) for that purpose, which will be a guide to him for making his incision; let it be carried to his left side and let him who standeth on the patient's right hand with his left hand lift up his scrotum; make your incision a finger's breadth from the fundament, on the left side of the Perinæum, not exceeding the bigness of your thumb, for it's afterward enlarged with the Dilator. If the Stone be found great, if it be possible let it be drawn out whole, if it cannot, it must bee broken: But after incision before you take out your Directory, thrust in the conductor upon the Directory to the very stone; after take out the Directory that so the yard may bee free. This done, thrust in the hamulus [hook] by the open side of the conductor; the conductor being drawn forth, then the stone to bee brought down by two of the fingers of the left hand put into the fundament, to bee caught by the hamulus, and so drawn out. Let some also crush the belly gently to further the falling of the stone to the neck of the bladder. After the great stones are drawn forth then with your spoon cleanse the bladder of the gravel and clotted blood. But this may be omitted if the Patient's strength will not bear it, the orifice having to be kept open. As soon as the bladder is discharged, let the ligature be unloosed and the Patient laid in his bed, apply to the wound a tent armed with the white of eggs and an astringent powder, which put to the very bladder, upon this a boulster dipped in the white of an egg, rose and plantain waters beat together. At night let it be dressed again, if hee have not urined, as before and so for three or four days, till danger of bleeding be past. A strict diet is to be observed and the belly kept open, and if the body be young and plethorick, a vein opened after the third day, which prevents most symptoms."

* The Lancet, vol. i. 1904, p.

